# An Exploratory Study: Can Native T1 Mapping Differentiate Sarcoma from Benign Soft Tissue Tumors at 1.5 T and 3 T?

**DOI:** 10.3390/cancers16223852

**Published:** 2024-11-17

**Authors:** Laura Dupont, Bénédicte M. A. Delattre, Marta Sans Merce, Pierre Alexandre Poletti, Sana Boudabbous

**Affiliations:** Diagnostic Department, Radiology Unit, Geneva University Hospital, 1205 Geneva, Switzerland; laura.dupont@chuv.ch (L.D.); benedicte.delattre@hug.ch (B.M.A.D.); marta.sansmerce@hug.ch (M.S.M.); pierre-alexandre.poletti@hug.ch (P.A.P.)

**Keywords:** T1 mapping, sarcoma, benign tumor, magnetic resonance imaging, 1.5 T, 3 T, differential diagnosis

## Abstract

**Background/Objectives**: T1 relaxation time has been shown to be valuable in detecting and characterizing tumors in various organs. This study aims to determine whether native T1 relaxation time can serve as a useful tool in distinguishing sarcomas from benign tumors. **Methods**: In this retrospective study, patients with histologically confirmed soft tissue sarcomas and benign tumors were included. Only patients who had not undergone prior treatment or surgery and whose magnetic resonance imaging (MRI) included native T1 mapping were considered. Images were acquired using both 1.5 T and 3 T MRI scanners. T1 histogram parameters were measured in regions of interest encompassing the entire tumor volume, as well as in healthy muscle tissue. **Results**: Out of 316 cases, 16 sarcoma cases and 9 benign tumor cases were eligible. The T1 values observed in sarcoma did not significantly differ from those in benign lesions in both 1.5 T and 3 T MRIs (p1.5T = 0.260 and p3T = 0.119). However, T1 values were found to be lower in healthy tissues compared to sarcoma at 3 T (*p* = 0.020), although this difference did not reach statistical significance at 1.5 T (*p* = 0.063). At both 1.5 T and 3 T, no significant difference between healthy muscle measured in sarcoma cases or benign tumor cases was observed (p1.5T = 0.472 and p3T = 0.226). **Conclusions**: T1 mapping has the potential to serve as a promising tool for differentiating sarcomas from benign tumors in baseline assessments. However, the standardization of imaging protocols and further improvements in T1 mapping techniques are necessary to fully realize its potential.

## Simple Summary

This study explored whether T1 relaxation time, a quantitative measurement taken during MRI scans that reflects tissue properties, can help differentiate between sarcomas and benign tumors in soft tissues. MRI scans of patients who had confirmed cases of either sarcomas or benign tumors and had not received prior treatment were analyzed. T1 values in both the tumors and surrounding healthy tissue were acquired with 1.5 T and 3 T MRI scanners. The results showed that sarcomas tended to have higher T1 values than benign tumors, although this difference was not always statistically significant. However, T1 values were notably lower in healthy tissues compared to sarcomas, especially at 3 T. While the study suggests that T1 mapping might help in distinguishing sarcomas from benign tumors, more standardized protocols and further research are needed to improve the accuracy of this technique.

## 1. Introduction

Soft-tissue sarcomas are rare cancers typically presenting as painless, enlarging masses in nearly all areas of the body. Diagnosis involves advanced imaging and confirmation through biopsy. The primary treatment is surgery, possibly supplemented by radiation therapy or chemotherapy. However, the treatment lacks a standardized protocol, and management depends on different factors such as tumor size, grade, and patient condition. The prognosis for low-grade sarcoma is excellent (90% 5-year survival rate), while it is more reserved for high-grade sarcoma (5-year survival rate of 50–70%). Therefore, accurate and early diagnosis is crucial [1].

Magnetic resonance imaging (MRI) is a cornerstone in the characterization and evaluation of soft tissue tumors [2,3,4], as well as in the early screening of sarcomas [5,6]. Diffusion-weighted imaging (DWI) and perfusion and dynamic contrast-enhanced (DCE) MRI have been utilized to differentiate benign from malignant tumors and to predict sarcoma grading [6,7,8], as well as response to treatment [9,10,11]. DCE MRI sequences assess the perfusion of sarcomas, and changes in sarcoma perfusion parameters have been correlated with genetic subtypes, as well as with the degree and grade of necrosis [12]. These findings can be compared with DWI results where advanced techniques such as intravoxel incoherent motion (IVIM) and diffusion kurtosis imaging (DKI) were found to be associated with histopathological findings [13,14,15]. However, perfusion analysis of this heterogeneous group of rare tumors remains challenging [16,17,18,19].

Native T1 mapping is employed to convert signal intensity measurements into contrast agent concentration curves within tissues. T1, also called the longitudinal relaxation time, is measured in milliseconds (ms) and reflects intrinsic properties of tissues. T1 relaxation time has been shown to be of interest in detecting and characterizing tumors in various organs. For example, in cardiac imaging, T1 was found to be elevated in fibrotic tissues, correlating directly with biopsy-proven fibrosis [20]. In brain tumors, Piper et al. demonstrated that T1 was significantly higher in low-grade gliomas compared to meningiomas [21]. In breast imaging, high-grade invasive ductal carcinomas exhibited longer T1 relaxation times than low-grade tumors [22]. Additionally, T1 mapping has been successfully applied to differentiate phyllodes tumors from fibroadenomas in the breast, with a sensitivity of 0.89 and 1, respectively, as phyllodes tumors demonstrate longer T1 relaxation times [23]. T1 mapping has also been used to distinguish between mucinous and non-mucinous rectal adenocarcinomas [24] and between benign prostate tissue and prostate carcinoma [25]. Furthermore, although few cases concerning musculoskeletal applications have been published, Baidya et al. demonstrated that T1 could serve as a marker of chemotherapy response in osteosarcomas [26].

It is important to note that T1 relaxation time can vary according to different parameters, most notably magnetic field strength. For musculoskeletal tumors, T1 has been shown to increase by 20% at 3 T compared to 1.5 T [27]. Given the ability of T1 mapping to differentiate malignant tumors from benign tissues in various organs, investigating whether T1 values could distinguish benign soft tissue tumors from sarcomas would be highly valuable.

The aim of our study was to determine whether native T1 relaxation time could be used to differentiate histologically proven sarcomas from benign soft-tissue tumors using appropriate DCE protocols.

## 2. Materials and Methods

### 2.1. Patient Inclusion Criteria

A retrospective survey was conducted to select appropriate cases for the study. The database of all patients referred to the multidisciplinary sarcoma board was examined. Histological characterization of the lesion was required to be available. Only MRI examinations conducted prior to any biopsy, treatment, or surgery were included. Furthermore, MRI acquisitions needed to contain a native T1 map. This retrospective study was approved by the local institutional review board and conducted in accordance with the principles of the Helsinki Declaration. Informed consent from patients was waived due to the retrospective nature of the study.

### 2.2. MRI Protocol

Regarding the MRI systems, 1.5 T and 3 T scanners from two different vendors (Philips, Best, Netherlands, and Siemens, Erlangen, Germany) were employed. The MRI protocol designed for all patients included pre- and post-injection sequences to enhance the visualization and characterization of the lesion. Prior to injection, T1-weighted (T1w) turbo spin echo (TSE) sequences and T2-weighted (T2w) TSE sequences with fat suppression (preferably using Short TI-Inversion Recovery (STIR)) or proton-density-weighted TSE with fat suppression were performed in one or two anatomical planes. A T1 map was acquired using a three-dimensional (3D) spoiled gradient echo sequence with fat saturation and varying flip angles (4° and 8°). The same sequence with a flip angle of 10° was utilized during contrast injection to observe wash-in and wash-out curves, lasting for 5 min, with the dynamic scan time maintained below 15 s. Following contrast injection, one or two anatomical planes were acquired using T1w TSE sequences with fat suppression. Each sequence was adapted to the area of interest. Table 1 summarizes the parameters of the T1 mapping sequence.

### 2.3. Image Analysis

On the Philips systems, native images from the T1-weighted 3D spoiled gradient echo sequence with fat saturation and two flip angles were transferred via a secured internal node to the Olea software SP28 (Olea, La Ciotat, France), where T1 maps were generated using the relaxometry module. On the Siemens system, the T1 map was generated online from the console. The T1 mapping images were transmitted to our picture archiving and communication system (PACS) for archiving. The Osirix DICOM viewer (Bernex, Switzerland) was utilized to perform measurements on the T1 maps. Regions of interest (ROIs) were delineated on the T1 maps within the lesion, encompassing the entire lesion volume. For healthy muscle, measurements were taken across five consecutive slices (for small corresponding lesions) or ten consecutive slices (for larger lesions). All ROIs were reviewed by a senior musculoskeletal radiologist. Corresponding mean, median, skewness, and kurtosis of the T1 relaxation times were reported.

### 2.4. Statistics

The data extracted from the images were analyzed using Python software (Python Software Foundation, Python Language Reference, Version 3.8.5, available at http://www.python.org, accessed on 1 September 2024). Two different homogeneity tests were applied for inter-group and intra-group comparisons. The comparison of parameters between benign tumors and sarcomas was performed using the Mann–Whitney U test, while the comparison of benign tumors or sarcomas against healthy tissues utilized the Wilcoxon test.

## 3. Results

### 3.1. Patient Inclusion

**Figure 1:**
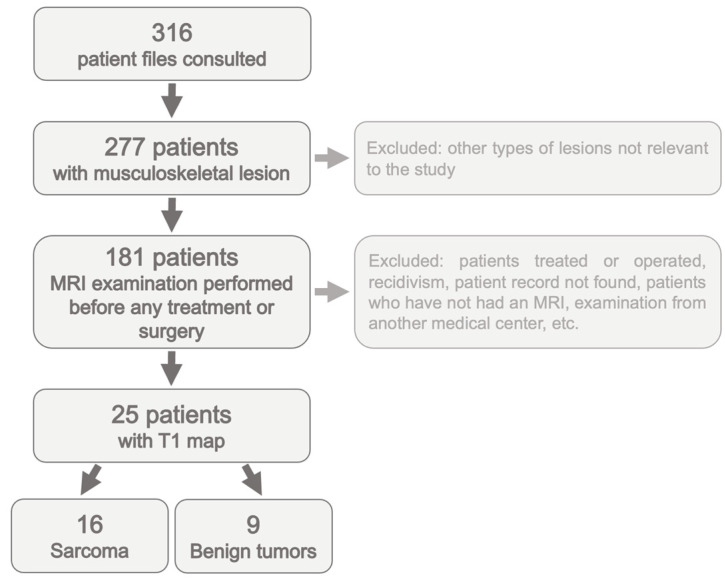
Patient inclusion diagram.

Out of 316 patients referred to our tertiary center with soft tissue tumors, only 16 patients with confirmed sarcomas and 9 patients with histologically confirmed benign tumors were included in the study; see the patient inclusion diagram in Figure 1. The types of sarcomas and benign tumors are summarized in Table 2.

### 3.2. T1 Values in Sarcomas and Benign Tumors

**Figure 2:**
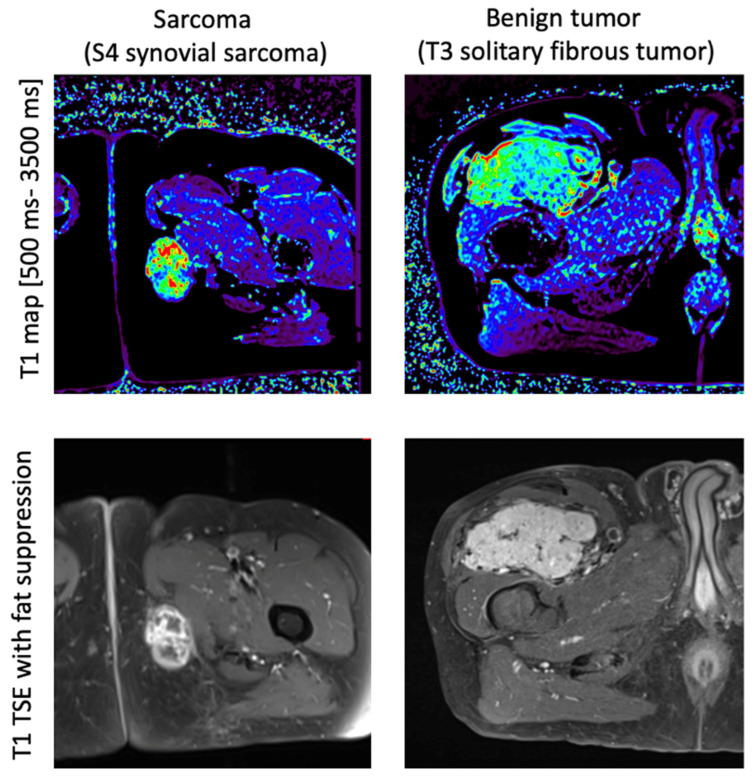
Case example for sarcoma and benign tumor. Upper line: T1 relaxation maps (scale 500–3500 ms) for a synovial sarcoma (**left**) and benign solitary fibrous tumor (**right**). Bottom line: T1w with fat suppression for corresponding slice.

**Figure 3:**
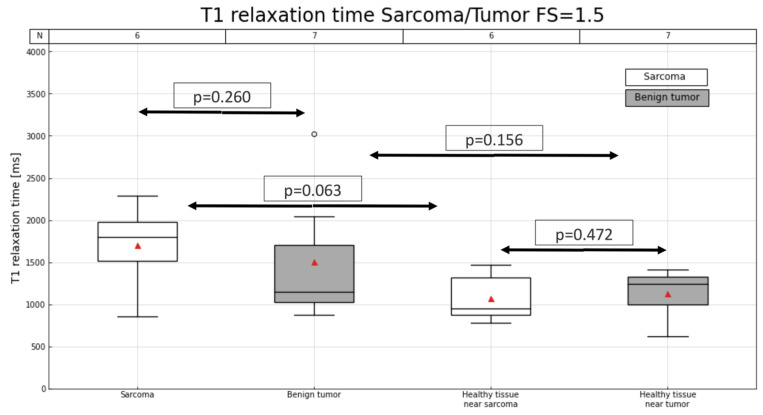
T1 relaxation time (ms) in lesion and in muscle for patients with sarcoma or benign tumor for a magnetic field of 1.5 T. Number of patients in each group is indicated (N) at the top of the graph. Red triangles represent mean T1.

**Figure 4:**
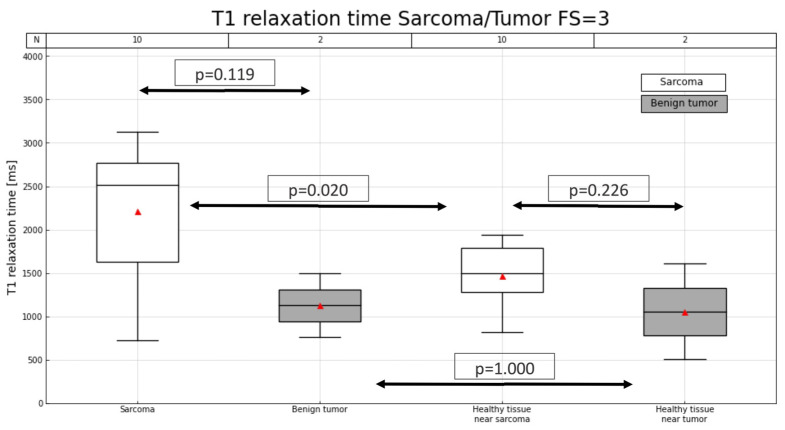
T1 relaxation time (ms) in lesions and in muscles for patients with sarcoma or benign tumor for a magnetic field of 3 T. Number of patients in each group is indicated (N) at the top of the graph. Red triangles represent mean T1.

An example of a T1 map involving sarcomas and benign tumors is shown in Figure 2, while Figure 3 and Figure 4 illustrate the distribution of T1 relaxation times in sarcomas and benign tumors, as well as the measurements in healthy muscle for comparison. At 3 T, lower T1 values were observed in healthy muscle compared to sarcomas (p3T = 0.020), while this difference was not significant at 1.5 T (p1.5T = 0.063). Additionally, T1 values in healthy muscle did not significantly differ from those in benign tumors (p1.5T = 0.156 and p3T = 1). Additionally, no significant differences in T1 values were observed between healthy muscle tissue from sarcoma and benign tumor cases (p1.5T = 0.472 and p3T = 0.226). Table 2 provides detailed information on the T1 relaxation measurements across all patients.

### 3.3. T1 Histogram Parameters in Sarcomas and Benign Tumors

The skewness and kurtosis of T1 relaxation times were also measured, with the histogram parameters summarized in Table 3. Different tendencies were observed at 1.5 T and 3 T. Specifically, skewness was significantly lower in benign tumors compared to sarcomas at 1.5 T, while the opposite trend was observed at 3 T. Additionally, at 1.5 T, skewness was significantly lower in muscle tissues of patients with benign lesions compared to those with sarcomas. Kurtosis showed a large standard deviation in the sarcoma group at both 1.5 T and 3 T, affecting both lesions and healthy areas.

## 4. Discussion

Despite the limited number of included cases, to the best of our knowledge, this study is the first to focus on the added value of T1 mapping for soft tissue tumors. At both 1.5 T and 3 T, we observed higher (though not statistically significant) T1 mean values in sarcoma lesions compared to healthy muscles, which aligns with the observations made by Baidya et al. [26]. The T1 values reported by Baidya were of the same order of magnitude but lower than our own findings. The values at 1.5 T reported by these authors were 831 ms for sarcomas and 683 ms for muscles, compared to 1701 ms and 1072 ms, respectively, in our study.

Other studies have reported similar findings, showing higher T1 values in phyllodes tumors compared to fibroadenomas [23]. Additional researchers have demonstrated that T1 relaxation times are higher in malignant breast tumors compared to benign ones [28].

Our T1 values were quite similar to those measured by Stanisz et al. [29]. According to these authors, at 1.5 T, normal T1 relaxation values in skeletal muscles were recorded at 1008 ms, while we obtained values of 1072 ms and 1131 ms in patients with sarcomas and benign tumors, respectively. At 3 T, Stanisz reported a T1 of 1412 ms, while we obtained values of 1470 ms and 1056 ms for sarcomas and benign tumors, respectively (the latter group consisting of only two patients). This concordance supports the use of T1 relaxation times in muscles as a baseline for comparison with soft tissue tumor values.

Several methods are currently available for estimating T1 mapping. While the gold standard involves an inversion recovery spin echo sequence, this technique is time-consuming and thus not clinically practical. The variable flip angle method has proven accurate and robust but is sensitive to B1 inhomogeneities at field strengths greater than 1.5 T, requiring multiple flip angles to provide robust T1 estimations [30]. T1 mapping using the dual flip angle method has been shown to be both efficient and fast and is frequently applied in the context of dynamic contrast-enhanced (DCE) imaging. However, depending on the combination of flip angles chosen, significantly different T1 estimates may result [31].

In our study, we used only two flip angles, which is suboptimal for accurately estimating T1 values. This likely affected the quality of our measurements. For example, in the study by Baidya et al. [26], four flip angles were used, but the sequence acquisition time ranged from 12 to 15 min, whereas each of our sequences required less than 1 min. Consequently, there is significant potential for improvement in our measurements should T1 mapping be explored as a potential marker in future studies.

In our study, the results were separated based on the two different field strengths, as it is well known that T1 relaxation values depend on resonance frequency [29]. In biological tissues, T1 values have been shown to increase with higher field strength, a trend that was also observed in our measurements. Skewness and kurtosis results could indeed be promising markers, as Baidya et al. [26] demonstrated that skewness was higher in healthy tissues than in sarcomas. Another recent study showed that, among other factors, ADC skewness was associated with the expression of hypoxia-inducible factor-1 alpha (HIF-1α), a key regulator of oxygen homeostasis in sarcoma [32]. However, in our study, we observed higher skewness values for sarcomas compared to healthy tissues at 1.5 T, with the opposite trend at 3 T. This discrepancy may be explained by the retrospective design of the study. Two different systems were used with different acquisition protocols for T1 mapping sequences, tailored to each patient’s area of interest. This likely introduced heterogeneity in the signal-to-noise ratio within the images, which may have affected the histogram parameters, making it difficult to separate this effect from the intrinsic T1-value heterogeneity within the lesions.

Radiomic analysis has been shown to greatly improve lesion characterization when applied to MR images. For example, in bladder tumors, it is excellent at discriminating between low-grade and high-grade tumors, as well as differentiating muscle-invasive from non-muscle-invasive cancer [33]. It can also be successfully applied to T1 mapping of rectal adenocarcinoma to discriminate between mucinous and non-mucinous adenocarcinoma, as well as to differentiate low-grade from high-grade lesions [34]. This is certainly a promising tool that merits further investigation; however, we believe that the MRI protocol should first be standardized, as it has been shown that radiomic features are highly dependent on MRI field strength, the manufacturer, and acquisition parameters [35].

Our study has several limitations, the first being the small number of patients included in each group. The inclusion of “native” soft tissue tumors using the same T1 mapping protocol was the primary factor contributing to the small group size. Furthermore, the heterogeneity in acquisition techniques, with different MRI systems and varying signal-to-noise ratios obtained for each patient, likely impacted the results. The T1 map sequence parameters were adjusted for each patient based on tumor size, location, and the receiver coil used. In future studies, this should be more standardized with predefined sequences. Due to the retrospective nature of our study, we were limited to using the data and protocols already available. However, given the promising trends observed, we strongly believe that T1 mapping could be improved by incorporating additional flip angles or using other sequence types, which, while slower, may yield more robust results. Due to these limitations, we were unable to establish a cut-off for T1 values to differentiate sarcomas from benign tumors. Additionally, we did not investigate whether T1 values correlated with tumor grading or histology due to the limited number of cases. The absence of low-grade tumors further prevents us from drawing conclusions about this subgroup from our study. The small cohort size also precluded detailed analysis across different subtypes. A larger, more homogeneous cohort would allow for more granular analysis, especially as sarcomas represent a diverse group of tumors with potentially distinct T1 behaviors. This limitation underscores the need for future studies with expanded sample sizes to provide deeper insights into subtype-specific characteristics.

## 5. Conclusions

In conclusion, our study demonstrated higher T1 relaxation values in sarcomas compared to healthy muscles and benign lesions. Despite the non-significance of our results, the data suggest that T1 relaxation times prior to contrast injection are worth considering as an additional marker for lesion characterization, potentially aiding radiologists in tumor evaluation. T1 mapping appears to be a promising tool for differentiating sarcomas from benign tumors during baseline assessments, as has been demonstrated in other organs. However, it is essential to account for technical aspects to optimize its use in sarcomas and establish it as a valuable additional marker for radiologists.

## Figures and Tables

**Table 1:** Details of sequence parameters for T1 maps of patients.

Patient	Category	Voxel Reconstruction Size	Acquisition Time per Flip Angle	Flip Angle (°)	TE/TR (ms)	Number of Slices	Magnetic Field Strength (T)	MRI System
S1	Sarcoma	0.35 × 0.35 × 3 mm	16 s	4/8	3.6/7.4	19	3	Philips
S2	Sarcoma	1.14 × 1.14 × 3 mm	4 s	4/8	1.4/3.7	20	1.5	Siemens
S3	Sarcoma	0.9 × 0.9 × 3.5 mm	12 s	4/8	2/4.4	40	1.5	Siemens
S4	Sarcoma	1.1 × 1.1 × 4 mm	10 s	4/8	1.9/4.2	40	1.5	Siemens
S5	Sarcoma	1.6 × 1.6 × 3.5 mm	11 s	4/8	1.6/4.7	40	3	Siemens
S6	Sarcoma	1.3 × 1.3 × 3 mm	11 s	4/8	1.6/4.7	36	3	Siemens
S7	Sarcoma	0.75 × 0.75 × 4 mm	13 s	4/8	1.8/5.3	36	3	Siemens
S8	Sarcoma	1.8 × 1.8 × 3 mm	13 s	4/8	1.5/4.9	36	3	Siemens
S9	Sarcoma	0.35 × 0.35 × 3 mm	17 s	4/8	3.7/7.5	68	3	Philips
S10	Sarcoma	1.1 × 1.1 × 3 mm	13 s	4/8	1.7/4.6	36	3	Siemens
S11	Sarcoma	0.9 × 0.9 × 3.5 mm	8 s	4/8	1.9/4.3	40	1.5	Siemens
S12	Sarcoma	0.73 × 0.73 × 3 mm	18 s	4/8	3.4/6.9	44	3	Philips
S13	Sarcoma	0.8 × 0.8 × 3.4 mm	17 s	4/8	3.6/7.4	44	3	Philips
S14	Sarcoma	1.1 × 1.1 × 3 mm	13 s	4/8	1.7/4.6	36	3	Siemens
S15	Sarcoma	0.78 × 0.78 × 3 mm	12 s	4/8	1.6/3.3	25	1.5	Philips
S16	Sarcoma	0.89 × 0.89 × 3.5 mm	8 s	4/8	2.0/4.3	40	1.5	Siemens
T1	Benign tumor	1.5 × 1.5 × 4 mm	13 s	4/8	1.8/4.2	64	1.5	Siemens
T2	Benign tumor	1.15 × 1.15 × 3 mm	3.5 s	4/8	1.4/3.7	20	1.5	Siemens
T3	Benign tumor	1.0 × 1.0 × 4 mm	8 s	4/8	1.4/3.7	36	1.5	Siemens
T4	Benign tumor	1.2 × 1.2 × 4 mm	7 s	4/8	1.4/3.7	80	1.5	Siemens
T5	Benign tumor	0.8 × 0.8 × 3.4 mm	17 s	4/8	3.4/7.0	44	3	Philips
T6	Benign tumor	0.63 × 0.63 × 3 mm	8 s	4/8	1.9/5.4	18	3	Siemens
T7	Benign tumor	0.9 × 0.9 × 3.5 mm	8 s	4/8	2.0/4.3	40	1.5	Siemens
T8	Benign tumor	1.1 × 1.1 × 4 mm	9.8 s	4/8	1.9/4.2	40	1.5	Siemens
T9	Benign tumor	1.1 × 1.1 × 4 mm	13 s	4/8	1.9/4.2	64	1.5	Siemens

MRI = magnetic resonance imaging; TE = echo time; TR = repetition time.

**Table 2:** T1 relaxation time (ms) in lesions (mass) and muscles of all patients.

Patient	Diagnosis	Grade	Mean T1 in Lesion (ms)	T1 Standard Deviation in Lesion (ms)	Mean T1 in Muscle (ms)	T1 Standard Deviation in Muscle (ms)	Field Strength
S1	Undifferentiated Sarcoma	3	3312	434	1668	136	3
S2	Dermatofibrosarcoma protuberans	-	2092	638	1192	231	1.5
S3	Synovial sarcoma	2	810	147	728	122	1.5
S4	Synovial sarcoma	3	2766	1085	1022	216	1.5
S5	Pleomorphic sarcoma	3	3138	599	1435	460	3
S6	Leiomyosarcoma	2	2247	983	1586	257	3
S7	Myxoid fusocellular sarcoma	3	3132	336	3320	565	3
S8	Myxofibrosarcoma	3	2294	261	831	158	3
S9	Synovial sarcoma	2	3208	536	1175	259	3
S10	Pleomorphic sarcoma	3	3494	582	1832	374	3
S11	Fusiform cell sarcoma	2	1696	656	929	521	1.5
S12	Myxofibrosarcoma	3	1134	491	2180	484	3
S13	Leiomyosarcoma	2	694	89	1092	141	3
S14	Osteogenic sarcoma	3	2645	613	1357	193	3
S15	Osteogenic sarcoma	3	1831	2039	1574	491	1.5
S16	Leiomyosarcoma	-	3196	679	899	133	1.5
T1	Desmoid tumor	-	1246	275	1216	131	1.5
T2	Myxoma	-	3191	864	1266	220	1.5
T3	Solitary fibrous tumor	-	2021	375	1013	212	1.5
T4	Mesothelial cyst	-	814	424	1162	144	1.5
T5	Schwannoma	-	814	60	509	58	3
T6	Inflammatory pseudotumor	-	1651	254	1386	178	3
T7	Angiolipoma	-	750	572	853	119	1.5
T8	Desmoid tumor	-	1106	295	311	33	1.5
T9	Desmoid tumor	-	1206	227	280	45	1.5

**Table 3:** Summary of histogram parameters for all patients, separated by field strength.

		Sarcoma			Benign Tumor			*p*-Value Sarcoma-Benign Tumor
		Lesion	Healthy Muscle	*p*-Value	Lesion	Healthy Muscle	*p*-Value	
**1.5 T**	**Mean [ms] (mean ± SD)**	1701 ± 450	1072 ± 269	0.063	1502 ± 720	1131 ± 274	0.156	0.260
	**Median [ms]**	1802	950		950	1238		
	**N**	6			7			
	**Skewness (mean ± SD)**	3.22 ± 3.69	2.48 ± 1.25	1.000	0.58 ± 1.92	0.79 ± 0.64	0.375	0.022
	**Kurtosis (mean ± SD)**	31.00 ± 52.9	32.67 ± 39.24	0.844	5.94 ± 13.29	4.12 ± 3.84	0.578	0.195
**3 T**	**Mean [ms]**	2214 ± 809	1470 ± 358	0.020	1126 ± 367	1056 ± 550	1.000	0.119
	**Median [ms]**	2510	1499		1126	1056		
	**N**	10			2			
	**Skewness (mean ± SD)**	0.04 ± 0.87	0.94 ± 0.69	0.027	0.22 ± 0.20	1.26 ± 0.36	0.500	0.373
	**Kurtosis (mean ± SD)**	1.00 ± 2.53	4.92 ± 5.33	0.160	−0.71 ± 0.00	7.98 ± 4.13	0.500	0.053

## Data Availability

Data used in the study are included in the article, further inquiries can be directed to the corresponding author.
